# Fighting the infodemic: the 4 i Framework for Advancing Communication and Trust

**DOI:** 10.1186/s12889-023-16612-9

**Published:** 2023-08-30

**Authors:** Anne E. Sundelson, Amelia M. Jamison, Noelle Huhn, Sarah-Louise Pasquino, Tara Kirk Sell

**Affiliations:** 1 Johns Hopkins Center for Health Security, 700 E. Pratt Street, Suite 900, Baltimore, MD 21202 USA; 2 Department of Environmental Health and Engineering, Johns Hopkins Bloomberg School of Public Health, 615 N. Wolfe Street, Room E7527, Baltimore, MD 21205 USA; 3 Department of Health, Behavior and Society, Johns Hopkins Bloomberg School of Public Health, 615 N. Wolfe Street, Baltimore, MD 21205 USA; 4 Department of International Health, Johns Hopkins Bloomberg School of Public Health, 615 N. Wolfe Street, Baltimore, MD 21205 USA

**Keywords:** Misinformation, Disinformation, Infodemic, Fact check, Social media, Social-ecological model

## Abstract

**Background:**

The proliferation of false and misleading health claims poses a major threat to public health. This ongoing “infodemic” has prompted numerous organizations to develop tools and approaches to manage the spread of falsehoods and communicate more effectively in an environment of mistrust and misleading information. However, these tools and approaches have not been systematically characterized, limiting their utility. This analysis provides a characterization of the current ecosystem of infodemic management strategies, allowing public health practitioners, communicators, researchers, and policy makers to gain an understanding of the tools at their disposal.

**Methods:**

A multi-pronged search strategy was used to identify tools and approaches for combatting health-related misinformation and disinformation. The search strategy included a scoping review of academic literature; a review of gray literature from organizations involved in public health communications and misinformation/disinformation management; and a review of policies and infodemic management approaches from all U.S. state health departments and select local health departments. A team of annotators labelled the main feature(s) of each tool or approach using an iteratively developed list of tags.

**Results:**

We identified over 350 infodemic management tools and approaches. We introduce the 4 i Framework for Advancing Communication and Trust (4 i FACT), a modified social-ecological model, to characterize different levels of infodemic intervention: informational, individual, interpersonal, and institutional. Information-level strategies included those designed to amplify factual information, fill information voids, debunk false information, track circulating information, and verify, detect, or rate the credibility of information. Individual-level strategies included those designed to enhance information literacy and prebunking/inoculation tools. Strategies at the interpersonal/community level included resources for public health communicators and community engagement approaches. Institutional and structural approaches included resources for journalists and fact checkers, tools for managing academic/scientific literature, resources for infodemic researchers/research, resources for infodemic managers, social media regulation, and policy/legislation.

**Conclusions:**

The 4 i FACT provides a useful way to characterize the current ecosystem of infodemic management strategies. Recognizing the complex and multifaceted nature of the ongoing infodemic, efforts should be taken to utilize and integrate strategies across all four levels of the modified social-ecological model.

## Background

In today’s interconnected, digitalized world, it has become increasingly apparent that information about a public health event—particularly false or misleading information—can lead to negative health outcomes. The ongoing COVID-19 pandemic has reinforced this fact, prompting the World Health Organization (WHO) to declare a simultaneous “infodemic” or “overabundance of false or misleading information on COVID-19, which poses a grave threat to response efforts and public health” [1]. In the midst of this infodemic, researchers have uncovered associations between exposure to or belief in COVID-19-related misinformation (false or misleading information that is spread unwittingly by those who do not know it is false) and psychological distress, non-adherence to recommended mitigation measures, reduced intent to get vaccinated, and violence against healthcare workers [2–5]. Disinformation, or false information that is spread deliberately by those seeking to cause harm, is also a growing concern, as there is evidence that state actors may be using disinformation to fuel pernicious debates about public health issues in the United States (US), particularly vaccination [6].

The rise of social media and digital technologies has undoubtedly contributed to the infodemic. Indeed, researchers have found that on Twitter, false information travels faster and more widely than true information [7]. The mechanisms that underly the viral spread of false information on social media are complex and contested, but scholars have highlighted the role of platform algorithms and echo chambers (online environments in which individuals only see content that aligns with their pre-existing beliefs), both of which may facilitate selective exposure to (potentially false) information [8–10].

Once exposed, cognitive and psychological processes dictate whether an individual will believe false information or reject it. Unfortunately, human cognitive processing is subject to inherent biases that can make individuals vulnerable to misinformation/disinformation [11]. There is some evidence that individuals may be more prone to these biases when presented with information in a so-called “filter-bubble” (an algorithmically curated information environment) or echo chamber [12], further underscoring the role of social media in the propagation of false information. Mistrust—of governments, individuals in positions of authority, or institutions—has also been implicated in belief in/the spread of misinformation and disinformation [13, 14], as individuals with high levels of mistrust are likely to reject official information and seek out alternative explanations (which may take the form of conspiracy theories) [15].

While a large and growing body of research is dedicated to understanding both the mechanisms and impact of misinformation and disinformation, fewer efforts have sought to characterize the full spectrum of misinformation/disinformation management strategies [16]. Two recent analyses, for example, characterized only a subset of existing strategies, focusing mainly on psychological and cognitive interventions [17, 18]. Further, these analyses focused on false information more generally, without a specific focus on strategies for managing health-related misinformation/disinformation. Over the past several years, numerous organizations have developed tools and approaches to manage the spread of falsehoods and communicate more effectively in an environment of misleading health claims. The WHO, for example, ran a crowdsourced technical consultation in 2020 on infodemic management strategies, leading to the development of an infodemic management framework [19]. Other groups have developed more local and community-based approaches, including training trusted community messengers to disseminate accurate information about COVID-19 [20]. Additionally, researchers have crafted innovative interventions designed to refute or confer resistance to health-related misinformation/disinformation [21–23]. Taken together, these tools and approaches can serve as a resource for public health practitioners and those working in health communications, research, or policy, who will be faced with health-related misinformation and disinformation for years to come. However, because such approaches have not been systematically characterized, practitioners and policy makers are unlikely to be able to take full advantage of them when crafting their own infodemic management strategies.

The aim of this analysis was to characterize the current ecosystem of infodemic management strategies, allowing public health practitioners, communicators, researchers, and policy makers to gain an in-depth understanding of the tools and approaches at their disposal. Specifically, we sought to accomplish two goals: first, in an exploratory review, we identify existing tools and approaches for infodemic management, and second, through a qualitative content analysis of these tools and approaches, we develop a conceptual framework to characterize points of infodemic intervention. This work was conducted as part of a large multi-stage research project exploring effective public health communication strategies to utilize in an environment of false or misleading information and mistrust.

## Methods

### Search strategy

The research team utilized a multi-pronged search strategy to identify tools and approaches for combatting health-related misinformation and disinformation. First, a scoping review of academic literature indexed in PubMed, Scopus, and Web of Science was conducted using two sets of keywords: one relating to misinformation and disinformation and another relating to management of or solutions to misinformation and disinformation. The search strategy for each database can be seen in Table 1.

**Table 1:** Search strategy for scoping literature review

Search Sequence	PubMed	Scopus	Web of Science
1	"Disinformation"[Mesh]	TITLE-ABS-KEY(misinformation OR disinformation OR “fake information” OR “fake news” OR “conspiracy theor*” OR infodem* OR “false science” OR “misleading information” OR rumor*)	TS = (misinformation OR disinformation OR "fake information" OR "fake news" OR "conspiracy theor*" OR infodem* OR "false science" OR "misleading information" OR rumor* OR rumour*)
2	misinformation[tiab] OR disinformation[tiab] OR "fake information"[tiab] OR "fake news"[tiab] OR "conspiracy theor*"[tiab] OR infodem*[tiab] OR "false science"[tiab] OR "misleading information" OR rumor*[tiab]	TITLE-ABS-KEY(“digital health literacy” OR “health literacy” OR “science literacy” OR “tools against” OR “preventive measure*” OR “methods to address” OR “infodemic management” OR “manag* infodemic*” OR “information literacy” OR “eHealth literacy” OR “fact check*” OR solution OR “rumor track*” OR mythbust* OR debunk* OR prebunk* OR rebuttal)	TS = ("digital health literacy" OR "health literacy" OR "science literacy" OR "tools against" OR "preventive measure*" OR "methods to address" OR "infodemic management" OR "manag* infodemic*" OR "information literacy" OR "eHealth literacy" OR "fact check*" OR solution OR "rumor track*" OR mythbust* OR debunk* OR prebunk* OR rebuttal)
3	#1 OR #2	#1 AND #2	#1 AND #2
4	"Health Literacy"[Mesh]		
5	"digital health literacy"[tiab] OR "health literacy"[tiab] OR "science literacy"[tiab] OR "tools against"[tiab] OR "preventive measure*"[tiab] OR "methods to address"[tiab] OR combat*[tiab] OR countermeasure*[tiab] OR "infodemic management"[tiab] OR "managing infodemic*"[tiab] OR "information literacy"[tiab] OR "eHealth literacy"[tiab] OR "fact check*"[tiab] OR solution*[tiab] OR "rumor tracking"[tiab] OR mythbust*[tiab] OR debunk*[tiab] OR prebunk*[tiab] OR rebuttal[tiab]		
6	#4 OR #5		
7	#3 AND #6		

To expedite the scoping review, final search results from all three databases were filtered to exclude non-review articles, yielding a total of 413 reviews. These reviews were uploaded to Covidence, a literature review software program. Duplicates were identified and removed, yielding 313 unique reviews. AES then conducted title and abstract screening followed by full text review. Reviews were included in the final corpus if they were accessible online, available in English, and contained discussion of interventions or strategies for managing/combatting health-related misinformation and disinformation. Reviews were excluded if they were not written in English, were not accessible online, or did not contain discussion of interventions or strategies for managing/combatting health-related misinformation and disinformation. A total of 43 reviews were included in the final corpus. These reviews were re-read in full by AES, who extracted individual tools, strategies, or approaches for managing/combatting health-related misinformation/disinformation and added them to an Excel file.

Next, all members of the research team (AES, AMJ, NH, and SLP) conducted independent searches of grey literature, including publications, reports, and products accessible through web searches. This search was built around a deductive list of organizations involved in misinformation and disinformation management and health communications, including international and intergovernmental organizations, US-based federal agencies, non-governmental organizations, technology and media companies, non-profits, think tanks, and research centers. To supplement the above searches, the research team scanned all U.S. state health department websites for misinformation and disinformation management practices, policies, and tools. In addition to state health departments, the websites of the following large local health departments were also searched for misinformation and disinformation management tools and strategies: the New York City Department of Health and Mental Hygiene, the San Diego County Health Department, Public Health – Seattle and King County, the Baltimore City Health Department, and the Philadelphia Department of Public Health. Team members also engaged in organic searches to identify additional sources. Tools and approaches were added to the Excel file as they were discovered, with care taken not to add duplicates.

Similar search terms were used to search the gray literature and health department websites as those used in the scoping literature review, though many websites did not have advanced search functions. As such, individual keywords or phrases were often used to search for relevant tools and approaches (e.g., “infodemic management” or “misinformation”). All searches were conducted between October 2022 and January 2023.

Tools and approaches were included in the Excel file if they were focused on addressing misinformation or disinformation related to a health topic (broadly defined). Tools and approaches were not limited by date of development and included those that emerged prior to COVID-19 as well as those that were in development at the time the searches were conducted. Further, tools were not limited by geography or language, but as our research team is based in the US and speaks English, these tools are more prominent in the data.

### Qualitative data analysis

The main feature(s) of each tool or approach were labelled in Excel using an iteratively developed list of tags. The initial list of tags was informed by the scoping literature review and developed by AES. This list was refined by the research team through group discussions as new tools and approaches were identified. Tools/approaches were coded with relevant tag(s) by the same researcher who entered the tool/approach into the working Excel file. Each entry could be coded with up to 3 tags. The research team held weekly meetings to discuss any coding questions and to revise the tag list as necessary.

## Results

We identified over 350 tools and approaches for managing health-related misinformation and disinformation. Many of the tools did not distinguish between misinformation and disinformation and were designed to combat false information in general (disinformation turns into misinformation once it is believed and propagated by those who believe it, so it is not always necessary or even possible to distinguish between the two [24]).

**Fig. 1:**
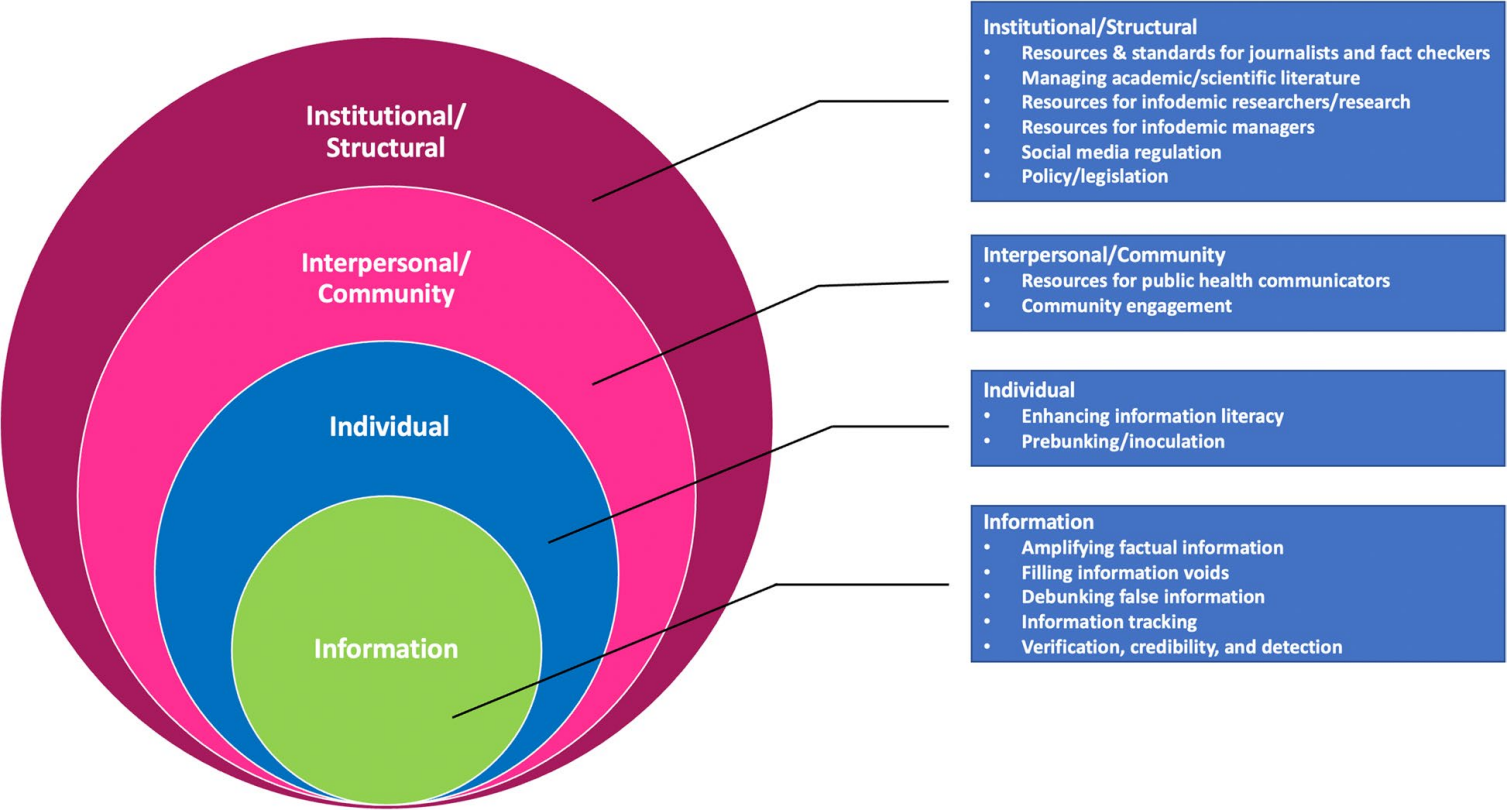
The 4 i Framework for Advancing Communication and Trust (4 i FACT) with types of tools and approaches

To characterize the infodemic management strategies identified in the search, we present the 4 i Framework for Advancing Communication and Trust (4 i FACT). The 4 i FACT, which is based on Bronfenbrenner’s ecological systems theory and the widely used social-ecological model (SEM) [25, 26], consists of four levels (information, individual, interpersonal/community, and institutional/structural), each of which contains a subset of the tags used to label individual strategies. A description of the tags in each level is shown in Fig. 1.

Each level of the 4 i FACT is described below, along with a description of the tags contained in each level and examples of the tools and approaches associated with each tag.

### Information

Tags in the information level were used to label tools or approaches that targeted information itself, including accurate information, false information, or lack of information (i.e., information voids).

#### Amplifying factual information

We identified 108 tools and approaches designed to disseminate or amplify accurate information or otherwise direct individuals to credible sources of information. These approaches often made use of social media to ensure accurate information reached as many people as possible. During the COVID-19 pandemic, for example, the Baltimore City Health Department launched a series of social media campaigns to ensure Baltimore residents had accurate and up-to-date information about the COVID-19 vaccines. The posts for these campaigns, which were written humorously using vernacular and graphics popular on social media, were designed to “go viral” [27]. Other organizations designed their approaches around social media to combat false information spread on these platforms. For example, “Dear Pandemic” is an ongoing effort to provide social media users with easy-to-understand, factual, and practical information about COVID-19 on Facebook and Instagram [28].

#### Filling information voids

We identified 50 tools/approaches designed to fill information voids. Some of these tools were chatbots that were programmed to answer common questions. VIRA, for instance, is a chatbot developed by the Johns Hopkins International Vaccine Access Center that uses artificial intelligence (AI) to answer common questions about the COVID-19 vaccines [29]. Other approaches relied on human interaction rather than AI. Several state health departments, for instance, including those in Minnesota [30], Georgia [31], and Illinois [32] ran telephone hotlines during the COVID-19 pandemic to answer residents’ questions. Search engine optimization was also used to fill information voids. The WHO and Google, for example, partnered during the COVID-19 pandemic to create an organized search results panel for anyone searching for information about COVID-19 online [33]. The search results panel directs Google users to credible sources of information like the WHO or CDC, thereby ensuring factual responses to search queries.

#### Debunking false information

We identified 100 tools/approaches designed to fact check or debunk circulating false information. Many of the tools with this tag were traditional fact-checking websites that provided lists of false claims and accompanying refutations or alternative explanations. Some of these websites were dedicated to either specific topics or specific sources of misinformation or disinformation. The #CoronaVirusFacts Alliance, for example, is a website containing a categorized database of fact-checked rumors about COVID-19 [34]. The EUvsDisinfo Database is a collection of debunked disinformation from pro-Kremlin sources. The database contains debunked claims on a variety of topics, including COVID-19, bioweapons, and other geopolitical issues [35].

#### Information tracking

We identified 44 tools/approaches designed to track circulating information, including false or misleading information. Many of these were social listening tools, which track conversations on social media and often rely on AI and machine learning (ML). The Early AI-Supported Response with Social Listening (EARS) Platform, for example, is a platform developed by the WHO that uses AI to search for COVID-19-related conversations and posts from major social media platforms, allowing users to gain an understanding of how individuals are talking about COVID-19 online [36]. Some of the tools and approaches with this tag facilitated reporting of misinformation. During the 2014–2016 Ebola outbreak, for example, a group of intergovernmental and academic organizations created DeySay, a rumor-tracking messaging system that allowed community members to report Ebola-related rumors via text message. The rumors reported through this system were used to inform relevant debunking materials, allowing public health communicators to refute misinformation in real time [37].

#### Verification, credibility, and detection

We identified 32 tools designed to detect false information or evaluate content or source credibility. These tools can be split into two broad categories: those designed to verify or rate sources of information, and those designed to confirm the authenticity of information by detecting manipulation or bot-like activity. An example of a tool that falls into the first category is Media Bias/Fact Check, which is a website that rates the bias and credibility of media sources and directs users to news pieces from the "least biased" sources [38]. An example of a tool in the second category is Botometer, which is an online tool that helps users determine whether specific Twitter accounts are likely to be bots [39]. Most of the verification, credibility, and detection tools were automated and relied on AI/ML.

### Individual

Tags in the individual level were used to identify tools and approaches designed to increase individual-level resiliency to misinformation and disinformation.

#### Enhancing information literacy

We identified 58 tools/approaches designed to encourage or teach individuals to think critically about the information they consumed, thereby reducing their susceptibility to false or misleading claims. Some of these approaches were focused on a single type or form of information, such as scientific or health-related information. The San Diego County Health Department, for example, developed an online resource (a webpage with links to other sites) informing users how to find credible scientific information about COVID-19 as well as how to critically evaluate scientific information about the disease [40]. Other tools and approaches were focused on digital or media literacy. For example, the non-profit New America is currently developing Cyber Citizenship, which is a collection of media and digital literacy resources for educators who are interested in helping their students build resilience to misinformation and disinformation online [41]. Other tools and approaches with this tag were designed to enhance information literacy more broadly. Sarah Blakeslee at California State University, Chico, for example, developed the CRAAP Test, which is a tool that helps individuals evaluate the credibility of a source of information based on its Currency, Relevance, Authority, Accuracy and Purpose (CRAAP) [42].

#### Prebunking/inoculation

Prebunking, also referred to as inoculation, is a strategy in which individuals are pre-emptively exposed to anticipated false information or common tactics used in misinformation and disinformation campaigns, making them (theoretically) less susceptible to misinformation and disinformation when they come across it [43, 44]. We identified 11 prebunking tools/approaches in this search, many of which were in gamified formats. For example, Go Viral! is an online game developed by the University of Cambridge, UK Cabinet Office, and the WHO. Players of the game learn how to create viral false content using common manipulation tactics. In doing so, they develop “psychological resistance” against future misinformation and disinformation campaigns [45].

### Interpersonal/community

Tags in the interpersonal/community level were used to label tools and approaches that were focused on communication and relationship or trust building at the interpersonal or community level.

#### Resources for public health communicators

These resources (of which we identified 62) were designed to enhance the credibility and efficacy of public health communication—particularly in the midst of mistrust and misinformation—and included messaging guidance, sharable materials, and toolkits. Many targeted traditional public health communicators, such as health department employees, physicians, or community health workers. The Public Health Communications Collaborative, for example, compiled a collection of toolkits, talking points, messaging, and graphics to help public health leaders communicate credibly and persuasively about COVID-19, along with other health topics [46]. Other resources targeted non-traditional public health communicators, including parents, teachers, and faith leaders. The Public Health Association of British Columbia, for example, partnered with CANVax to develop The COVID-19 Misinformation Toolkit for Kids (and Parents!) at Home, which is a guide for parents outlining how to discuss COVID-19 vaccines with their children [47]. In addition, in 2021, the Office of the U.S. Surgeon General released A Community Toolkit for Addressing Health Misinformation, which provides guidance to teachers, school administrators, healthcare professionals, community members, and faith leaders on understanding, identifying, discussing, and ultimately combatting health-related misinformation [48].

#### Community engagement

We identified 25 community engagement approaches. These approaches typically involved efforts to identify and train trusted messengers who could communicate accurate health information or encourage protective health-related behavior among members of their communities. For example, the nonprofit Vaccinate Your Family recently developed SQUAD™, which is a program that provides training and mentorship to individuals who want to become vaccine advocates in their communities [49]. Many of the approaches in this level were aimed at overcoming communication barriers (like lack of trust) among hard-to-reach, marginalized, or vulnerable populations. Live Chair Health, for example, is an organization that trains U.S. barbers in health education in order to close the life expectancy gap and overcome medical mistrust among Black men. Recently, they have been training barbers to discuss COVID-19-related issues with their clients, including vaccination [50]. Some of the community engagement efforts we identified were designed to (re)build trust in the healthcare system at large. The International Vaccine Access Center, for example, partnered with local community leaders and other organizations in Baltimore to counter vaccine myths and encourage members of the African American community to get vaccinated against COVID-19. The more overarching goal of the program, however, was to “build trust in both vaccination and the broader health system” [51].

### Institutional/structural

Tags in the institutional/structural level were often applied to tools or approaches that were designed to shift the burden of infodemic management from the “demand” side (i.e., focusing on information consumers) to the “supply” side (i.e., focusing on information purveyors).

#### Policy or legislation

We identified 33 regulatory or legislative approaches. These approaches can be divided into three categories. The first category consisted of efforts to regulate online content or otherwise hold individuals or companies criminally responsible for sharing false information online. In the US, for example, some have proposed changes to Sect. 230 of the Communications Decency Act, which protects online platforms from legal action based on the content shared by third parties. Proposed changes are intended to amend Sect. 230 by making online platforms liable for using their algorithms to promote the spread of health-related misinformation during a public health emergency [52].

The second category of regulatory and legislative approaches consisted of policies designed to enhance digital or media literacy. The government of Singapore, for example, recently released its Digital Readiness Blueprint, which is a national plan for increasing access to and use of digital technology as well as enhancing digital literacy among citizens. One of the aims outlined in the plan is to “strengthen focus on information and media literacy to build resilience in an era of online falsehoods” [53]. In the US, proposed legislation has included efforts to implement a national strategy for information/media literacy education and the development of a commission to oversee information/media literacy in schools [54].

The final category of policy/legislative approaches consisted of policies related to medical boards or licensure in the US. The Tennessee State Medical Board, for example, instituted a policy in 2021 that allows removal of medical licenses from physicians spreading misinformation about the COVID-19 vaccines [55].

#### Social media regulation

We identified 13 approaches or policies designed to regulate information on social media platforms. These efforts can be divided into two broad categories: soft content moderation and hard content moderation. Soft content moderation generally consisted of efforts to reduce the visibility or amplification of posts containing false information or efforts to alert individuals that certain posts may contain false content. Hard content moderation involved removal of posts or suspension of accounts propagating false information. Meta is an example of a social media company that has employed both soft and hard content moderation. During the COVID-19 pandemic, for example, Meta introduced a series of policies to combat misinformation and disinformation about the virus on Facebook, including using its algorithm to limit the spread of false information and removing posts or accounts responsible for repeatedly sharing misinformation [56].

The remaining institutional/structural-level tools consisted of capacity building tools for those working in health communications, public health, or infodemic research/management.

#### Managing academic/scientific literature

We identified 3 tools/approaches designed to help academics or public health professionals keep track of emerging or retracted scientific literature. The COVID Contents (CC) Initiative, for example, was an effort undertaken by the Istituto Superiore di Sanità (ISS) in Italy. During the height of the COVID-19 pandemic, ISS established a working group to sift through peer-reviewed papers and pre-prints on COVID-19. The working group compiled their findings into an open-access weekly report called Covid Contents, the aim of which was to provide health professionals with up-to-date and synthesized information about COVID-19 as it emerged [57].

#### Resources and standards for journalists/fact checkers

We identified 13 resources or standards for journalists/fact-checkers. Some of these were designed to ensure journalists had access to accurate information and adequate resources when reporting on public health emergencies. In 2020, for example, the International Center for Journalists, together with the International Journalists’ Network launched the Global Health Crisis Reporting Forum, now called the ICFJ Pamela Howard Forum on Global Crisis Reporting, which provided journalists with information about COVID-19 along with other resources to improve their coverage of the pandemic [58]. Other tools and approaches with this tag aimed to improve or bolster the fact checking industry. The International Fact Checking Network (IFCN) Code of Principles, for example, is an effort to promote fact checking in journalism and establish professional standards and codes of conduct for fact checkers across the globe [59].

#### Resources for infodemic researchers/research

We identified 9 tools/approaches designed to facilitate research on misinformation/disinformation and infodemiology. The Mercury Project, for example, is an effort to fund research that will help “combat the growing global threat posed by low Covid-19 vaccination rates and public health mis- and disinformation” [60].

#### Resources for infodemic managers

We identified 38 high-level resources for those managing misinformation and disinformation as public health, community, or industry leaders. Many of the tools and approaches with this tag consisted of frameworks, toolkits, or high-level guides outlining how to combat health-related misinformation/disinformation or infodemics more broadly. The U.S. Cybersecurity and Infrastructure Security Agency (CISA), for example, developed the COVID-19 Disinformation Toolkit, which provides information and guidance to state, local, tribal, and territorial officials on misinformation and disinformation related to COVID-19 [61].

## Discussion

While not comprehensive, the tools and approaches identified by the research team provide valuable insight into the current ecosystem of infodemic management strategies, which can be characterized using a modified social-ecological model with four levels. The tools/approaches in each level target important components and determinants of health-related misinformation and disinformation, including information itself, individual resiliency, communication and interpersonal/inter-community relationships and trust, and institutional and structural factors. However, each type of approach has accompanying strengths and weaknesses.

In terms of the information-level approaches, there are some practical considerations that are important to acknowledge. Findings from cognitive and psychological research suggest that human information processing is dictated largely by biases and heuristics [62], particularly in conditions of uncertainty [63]. If, for example, information provided to individuals is contrary to pre-established beliefs, such information (factual or not) may simply be dismissed in favor of alternative explanations, a phenomenon referred to as confirmation bias [64]. This bias not only makes individuals vulnerable to false information (particularly if such information conforms with their pre-existing beliefs), but also likely limits the impact of many of the information-level approaches in the database, including amplifying factual information, filling information voids, verification/credibility/detection, and debunking false information. Indeed, there is evidence that debunking false information is extremely challenging when such information aligns with individuals’ pre-existing beliefs [65]. The scale of false information also presents a practical challenge, as new rumors and claims constantly emerge. While the incorporation of artificial intelligence tools can support information-level interventions at scale, they introduce an additional set of complications and challenges associated with accuracy, interpretation, and the need for trained or experienced personnel [66].

In contrast to the information-level approaches, individual-level approaches are designed to encourage individuals to think more critically about information they come across, thereby helping them overcome some of the cognitive biases and heuristics that make them susceptible to false information in the first place. There is some evidence that such approaches can be effective. Prebunking interventions, for example, have been shown to reduce the likelihood that individuals will be persuaded by false information or share it with others [43–45, 67]. In addition, there is evidence that information literacy interventions can change the way individuals think about and evaluate the information they consume [68, 69]. However, in order for such interventions to have real-world impact, individuals must agree to be inoculated and/or undergo information literacy training. This could prove challenging, especially considering the ongoing politicization of public health. Enhancing individual-level resiliency will also need to be a continual process as increasingly savvy actors and misinformation campaigns continue to adapt and evolve. Finally, it should be noted that enhancing science literacy will not necessarily make individuals more trusting of information provided to them by scientists. On the contrary, improving individuals’ knowledge of the scientific process (and of the inherent uncertainties involved in scientific research) may cause them to be more skeptical of scientific information in general [70].

The communication and community engagement approaches identified in this search touch on one of the most important components of and contributors to misinformation and disinformation: lack of trust. By leveraging trusted community messengers and (re)building trust in the healthcare system, these approaches offer promising ways to overcome barriers to communication and reduce the spread and impact of false information. However, identifying messengers and establishing trust with certain communities—particularly those that have experienced marginalization or oppression—will require ongoing investment and resources. Indeed, scholars argue that community engagement should be thought of as a component of disaster preparedness in addition to response [71]. Moreover, community engagement and communication approaches will need to be tailored to the specific information needs of different communities. Information tracking tools may help identify such needs, as well as what kind of false information is circulating at a given time.

The institutional and structural-level approaches—particularly those relating to social media regulation and policy or legislation—are important given that they allow for a more supply-side approach to combatting misinformation/disinformation. Such approaches may be valuable because, as discussed above, cognitive biases make it difficult to prevent individuals from believing false information or to correct it once it has been seen. However, there may be unintended consequences associated with efforts to regulate the supply of false information. For example, there is evidence that flagging false content, a form of social media regulation, may make individuals more likely to believe that content that is not flagged is true [72]. This phenomenon, referred to as the implied truth effect, could be problematic if unflagged content is actually false. In addition, social media regulation could potentially increase conspiratorial beliefs or claims among those whose social media activity is limited by such regulation (i.e., shutting down accounts may prove to individuals that they are being lied to or that there is a conspiracy against them) [73, 74]. The very architecture of social media may also undermine efforts to contain misinformation, as financial incentives to keep users engaged continue to prioritize sensational content over more staid, but factual, claims. Structural approaches that require policy change may also be difficult to enact. In the US, political tensions have colored debates over social media policies. It should also be noted that legislative efforts to contain “misinformation” have been used to legally arrest and detain journalists and others around the world. In some instances, the arbiter of “truth” may be a government or administration that is hostile to claims that undermine its legitimacy.

Notwithstanding the challenges and limitations described above, each level of the 4 i FACT contains valuable approaches for managing and mitigating the effects of health-related misinformation and disinformation. Similar levels (information, population, system) have been identified in previous work on the evaluation of emergency risk communication [75], which suggests that social-ecological models offer a useful way to characterize points of intervention or evaluation of information-related processes during public health emergencies. Such models are likely useful because they reflect complex realities. Indeed, information (including false information) does not exist in a vacuum, but in a complex system of individuals, communities, and institutions. The 4 i FACT reflects this reality, offering possible points of intervention at each level of the system. However, given their associated limitations, interventions that target only one level of the system are unlikely to be effective on their own. As such, the most effective strategy for combatting health-related misinformation and disinformation will likely be one that is multi-faceted and stretches across multiple (or ideally all) levels of the 4 i FACT.

This research offers a characterization of infodemic management strategies that public health practitioners, communicators, and policy makers can use to guide current and future approaches. However, this study is subject to some limitations. The dataset developed for this study is not an exhaustive list of all past or existing misinformation and disinformation management strategies. Tools and approaches that were not discussed in academic or gray literature or that were not featured on U.S. state or selected large local health department websites were likely missed. Moreover, given that searches were conducted in English and only U.S. health department websites were searched, the database is unlikely to be representative of strategies at the international level. Finally, while efforts were taken to ensure tools and approaches were described using the most up-to-date and accurate information available, it is possible that some were misinterpreted or mischaracterized. Recognizing that any effort at a comprehensive list would be outdated as soon as it was compiled, the tagging system developed by the research team focuses on broader approaches that continue to resonate, even as the details of specific tools continue to evolve.

## Conclusions

The current ecosystem of infodemic management strategies can be characterized using a modified social-ecological model, the 4 i FACT, with four interconnected, nested levels: information, individual, interpersonal, and institutional. Public health practitioners, communicators, and policy makers can use this model, and the approaches contained within it, to inform current and future efforts to combat health-related misinformation and disinformation, which continue to pose a threat to public health. Given the complexity of the information environment and the fact that approaches in each level have associated strengths and limitations, efforts should be taken to utilize and integrate strategies across all four levels of the 4 i FACT. No single intervention can adequately address all levels of the infodemic, and any comprehensive approach to infodemic management must consider action across all levels.

## Data Availability

The datasets generated and analyzed during the current study are not yet publicly available as the research team is still exploring ways to display the data in a searchable format. In the meantime, data are available from the corresponding author on reasonable request.

## Abbreviations

WHO: World Health Organization
US: United States
4 i FACT: 4 I Framework for Advancing Communication and Trust
SEM: Social-ecological model
CDC: Centers for Disease Control and Prevention
NASEM: National Academies of Sciences, Engineering, and Medicine
AI: Artificial intelligence
ML: Machine learning
